# An Innovative Polymer-Based Electrochemical Sensor Encrusted with Tb Nanoparticles for the Detection of Favipiravir: A Potential Antiviral Drug for the Treatment of COVID-19

**DOI:** 10.3390/bios13020243

**Published:** 2023-02-08

**Authors:** Marwa F. B. Ali, Roshdy E. Saraya, Sami El Deeb, Adel Ehab Ibrahim, Baher I. Salman

**Affiliations:** 1 Department of Pharmaceutical Analytical Chemistry, Faculty of Pharmacy, Assiut University, Assiut 71526, Egypt; 2 Pharmaceutical Analytical Chemistry Department, Faculty of Pharmacy, Port-Said University, Port Said 42526, Egypt; 3 Institute of Medicinal and Pharmaceutical Chemistry, Teschnische Universität Braunschweig, 38106 Braunschweig, Germany; 4 Natural and Medical Sciences Research Center, University of Nizwa, Birkat Al Mauz, P.O. Box 33, Nizwa 616, Oman; 5 Pharmaceutical Analytical Chemistry Department, Faculty of Pharmacy, Al-Azhar University, Assiut Branch, Assiut 71524, Egypt

**Keywords:** Favipiravir, m-THB polymer, Tb nanoparticles, COVID-19, human plasma analysis

## Abstract

An innovative polymer-based electro-sensor decorated with Tb nanoparticles has been developed for the first time. The fabricated sensor was utilized for trace determination of favipiravir (FAV), a recently US FDA-approved antiviral drug for the treatment of COVID-19. Different techniques, including ultraviolet-visible spectrophotometry (UV-VIS), cyclic voltammetry (CV), scanning electron microscope (SEM), X-ray Diffraction (XRD) and electrochemical impedance spectroscopy (EIS), were applied for the characterization of the developed electrode Tb_NPs_@ poly m-THB/PGE. Various experimental variables, including pH, potential range, polymer concentration, number of cycles, scan rate and deposition time, were optimized. Moreover, different voltammetric parameters were examined and optimized. The presented SWV method showed linearity over the range of 10–150 × 10^−9^ M with a good correlation coefficient (R = 0.9994), and the detection limit (LOD) reached 3.1 × 10^−9^ M. The proposed method was applied for the quantification of FAV in tablet dosage forms and in human plasma without any interference from complex matrices, obtaining good % recovery results (98.58–101.93%).

## 1. Introduction

Favipiravir (FAV), 6-fluoro-3-hydroxy-2-pyrazinecarboxamide, is a promising antiviral pro-drug belonging to the RNA polymerase inhibitors. It was first introduced in Japan in 2014 as an anti-influenza agent, and it was also applied to treat other viruses [1]. In 2019, it was proven to be safe and effective against COVID-19, a global pandemic with outbreaks all over the world, as the World Health Organization (WHO) officially declared in March 2020. COVID-19 causes severe respiratory syndrome leading to a serious disease that affects different organs, including the kidneys, liver and central nervous system [2].

Although the pandemic crest has diminished lately due to several considerations, such as global vaccination and enhanced health awareness, however, its endemicity is said to be meaningless or at least only transitional. Expectations for infection spikes are high, which may be owed to the removal of restrictive measures as well as viral mutations [3,4]. Therefore, it was recommended to establish a sensitive, cost-effective and selective analytical method for the estimation of COVID-19 defense drugs such as FAV in biological fluids for routine drug quality control and further clinical studies’ monitoring. 

Although different analytical methods were published for the estimation of FAV, including spectrophotometric and spectrofluorimetric [5,6,7,8,9], thin layer chromatography [10,11], and liquid chromatography methods [5,7,12,13,14,15,16,17]. However, only a few voltammetric and electrochemical methods have been reported for the estimation of FAV till now [18,19,20,21,22,23]. Electrochemical techniques are currently more frequently used due to their simplicity, sensitivity, environment-friendly and ready availability. Besides, varying modifications of carbon-based electrodes have currently attracted considerable attention, where different nanomaterials [24,25] could be utilized for the detection of several analytes in complex matrices, such as pharmaceutical formulations and biological fluids. A pencil graphite electrode (PGE) is a working electrode that is commonly used currently. This can be attributed to its superb electrochemical properties like high conductivity, low cost, simplicity in modification and wide commercial availability [26,27,28]. Henceforth, it was recommended to establish a sensitive, selective and cost-effective electrochemical analytical approach for the estimation of FAV in human plasma for drug monitoring and further clinical studies.

Moreover, electro-polymerization has drawn great attention recently as one promising approach in electrochemistry [29]. Polymer-modified electrodes are used in the manufacturing of batteries or supercapacitors, organic light-emitting diodes and biosensors. The deposited polymers on the substrate surface own many properties like advanced electrical conductivity, redox mediating capabilities and specific adhesive and/or binding properties. These polymers are promising in electrode modification because it generally results in creating polymer film which is uniform and strongly adherent to the electrode surface [30]. Conducting polymers with extended π-π conjugated systems have been used in the synthesis and preparation of various electrochemical sensors [31,32,33] owing to their high conductivity and their redox properties. In addition, polymerization using polyhydroxy aromatic compounds has shown good stability, reproducibility, more active surface area and homogeneity in electrochemical deposition [34]. In the represented research, innovative polymer layers were created for the first time via utilizing phloroglucinol, a trihydroxy benzene compound (m-THB), which was electropolymerized in order to form electro-active layers on the surface of a solid surface of PGE.

Additional enhancement of the electrode activity and sensitivity was accomplished by the integration of metal nanostructures. In this article, we investigate the behavior of terbium nanocomposites. Terbium (Tb) is one of the lanthanide metals that has the ability to create more electronic charges or holes, efficiently generating an electron transfer pathway due to possessing two states of valence (Tb^3+^ and Tb^4+^). Hence, the incorporation of Tb^3+^ nanoparticles (Tb_NPS_) into any electrode modification is successfully enhancing the electronic conductivity, electrocatalytic activity, general electrochemical behavior, overall electrochemical performance and hence method sensitivity [35,36,37]. The incorporation of m-THB polymerized layers and Tb_NPS_ not only improves polymer characteristics but also provides excellent performance by increasing the active sites of the electrode surface utilizing these hybrid components.

Herein, a new strategy was introduced involving the design of the hybrid nanostructure composed of m-THB polymer layers over the PGE surface. Further, the incorporation of Tb_NPS_ overlaying the electro-active polymer layers was performed. The prepared electrode was characterized by UV-VIS spectrophotometry, scanning electron microscope (SEM), CV, SWV, and EIS methods. Besides, the modified sensor’s electrochemical performance was considered in monitoring the electro-oxidation of FAV in human plasma and tablets. The proposed SWV method is simple, highly sensitive and can be recommended for therapeutic drug monitoring (TDM) and various clinical laboratories for further pharmacokinetic studies.

## 2. Materials and Methods

### 2.1. Materials

FAV (purity = 99.0%) authentic standard was obtained from EIPICo. (Tenth of Ramadan City, Egypt). Avipiravir^®^ tablets (200 mg of FAV per tablet) were supplied from EVA Pharm (Giza, Egypt).

All chemicals and solvents were of analytical grades. Terbium chloride (III) hexahydrate (TbCl_3_ · 6H_2_O), phloroglucinol (m-trihydroxy benzene, m-THB), potassium chloride, potassium ferrocyanide were obtained from Sigma–Aldrich. HPLC grade methanol was obtained from Sigma–Aldrich Co. (St. Louis, MO, USA). Phosphate buffer (0.1 M, pH 4.0–9.0) was prepared using sodium dihydrogen phosphate and di sodium hydrogen phosphate (El-Nasr Pharmaceutical Chemicals Co., Cairo, Egypt); pH was adjusted by 0.2 M NaOH. Rotring (HB) pencil leads (0.5 mm × 60 mm) were purchased from a local market.

Blank human plasma samples (fresh frozen plasma, B-Rh+) were supplied from Assiut University Hospitals Blood Bank (Assiut, Egypt).

### 2.2. Instruments

Voltammetric measurements were done using a Princeton VersaSTAT MC (VersaSTAT 3, model RE-1, Princeton Applied Research, AMETEK, Easttown Township, PA, USA), which consists of a 3-electrode electroanalytical cell (Ag/AgCl, 3 M KCl reference electrode, a platinum wire auxiliary electrode and PGE either bare or modified was a working electrode). Ultrasonic cleaner (Cole-Parmer, Chicago, IL, USA), Sartorious handy balance-H51 (Hannover, Germany), Hanna pH meter HI 4222 (Hanna Instruments Brazil, Sao Paulo, Brazil), Boeco laboratory centrifuge U-320® (, Hamburg, Germany), FALC vortex-MIX (FALC Instruments, Treviglio, Italy), and Scanning electron microscope; SEM (JEOL JSM-5400 LV instrument, Oxford, MO, USA) were used. UV–VIS spectra of the modified layers were recorded using a UV-visible spectrophotometer (UV-1601PC, Shimadzu, Kyoto, Japan) with 1.0 cm quartz cells. The X-ray powder diffraction was measured using a Philips X-ray PW 1710 with Cu Ka radiation (l ¼ 1.5405 °A) with 40 kV and 30 mA. The scanning rate was maintained at 0.06 scans per minute, in the 2q range of 4–80.

### 2.3. Standard and Reagent Solutions

A standard solution of FAV (5 × 10^−6^ M) was prepared in double distilled water. Phloroglucinol (m-trihydroxy benzene; m-THB) solution (32 × 10^−3^ M) and Tb (III) solution (500 × 10^−3^ M) were formed in double distilled water. Further dilutions were done using the same solvent to investigate the optimum concentration for the polymerization of m-THB and the optimum concentration for the electro-deposition of Tb. 

### 2.4. Fabrication of Tb_NPS_@ Poly m-THB/PGE

To fabricate the presented electrode, the PGE surface was first washed with double distilled water before use. Further, an electro-polymerization of m-THB was established using 8 × 10^−3^ M of m-THB solution in phosphate buffer (0.1 M, pH 7) through multiple cyclic voltammetry for 10 cycles using a potential range of −0.9–+1.75 V and scanning rate of 0.1 Vs^−1^. The electrode was denoted as poly m-THB/PGE. Furthermore, the electrode was submerged in an electrochemical cell having Tb (III) chloride solution (125 × 10^−3^ M), which was electro-deposited using a potential at −1.2 V for 80 s. The fabricated Tb_NPs_@ poly m-THB/PGE electrode was further characterized and checked by UV-Visible spectrophotometry, CV, SEM and EIS.

### 2.5. Analytical Procedures for Estimation of FAV

#### 2.5.1. General Analytical Procedure

An appropriate volume of FAV (5 × 10^−6^ M) standard or sample solution was added into an electrochemical cell filled with 0.1 M phosphate buffer (pH 7) as a supporting electrolyte. The electrochemical performance of FAV using bare and modified PGE was studied using CV and SWV techniques. The experimental variables of the developed method in terms of m-THB polymerization, Tb_NPs_ electro-deposition and the optimum electrolyte pH were studied. Moreover, various SWV parameters were studied, including deposition time, frequency, initial potential and step and pulse height.

#### 2.5.2. Procedure for Estimation of FAV in Tablets

Ten Avipiravir^®^ tablets (200 mg per tablet) were weighed, crushed finally and thoroughly mixed. Then, an adequate weight equivalent to 10.0 mg FAV was transferred into a volumetric flask and dissolved into 50 mL of double distilled water. The solution was sonicated for about 20 min, followed by filtration, then the volume was made up to 100 mL with double distilled water to get a concentration of 100 µg mL^−1^. The developed SWV method was then carried out as mentioned above.

#### 2.5.3. Estimation of FAV in Human Plasma

In a centrifuge tube, 1.0 mL of human plasma was spiked with an adequate amount of FAV solution, and the volume was then furtherly made up to 10 mL using methanol. The mixture was vortexed for 30 seconds and then centrifuged for 35 min (3500 rpm). The resultant supernatant was collected, and appropriate volumes were added to the electrochemical cell. The voltammograms of SWV were recorded for FAV using the modified Tb_NPs_@ poly m-THB/PGE electrode under the optimum experimental conditions. A blank measurement was established in the same way but without the drug. In addition, required dilutions from this supernatant were made using the selected supporting electrolyte. The study was performed following the relevant faculty laws and guidelines, as well as the research ethics committee.

### 2.6. Characterization of Tb_NPs_@ Poly m-THB/PGE

The developed electrode was morphologically characterized using SEM and the modified polymer layers. Besides this, the XRD and UV–VIS spectra of the modified polymer layers and Tb_NPS_ were studied. In addition, the ESI method for bare and Tb_NPs_@ poly m-THB/PGE was examined.

### 2.7. Validation Data

The proposed SWV method was validated following the ICH guidelines [38] for linearity range, the limit of detection (LOD), the limit of quantification (LOQ), accuracy and precision. The LOD and LOQ values were determined using formulas 3 σ/S and 10 σ/S, respectively, where σ represents the standard deviation of the intercept and S is the slope of the related regression equation. The electrochemical technique’s precision was examined using three different concentrations within the calibration range within the same day (intra-day precision) and across three successive days (inter-day precision). The mean values of relative standard deviations (RSD%) of the results were calculated.

## 3. Results

### 3.1. Preparation of Tb_NPS_@ Poly m-THB/PGE

**Figure 1:**
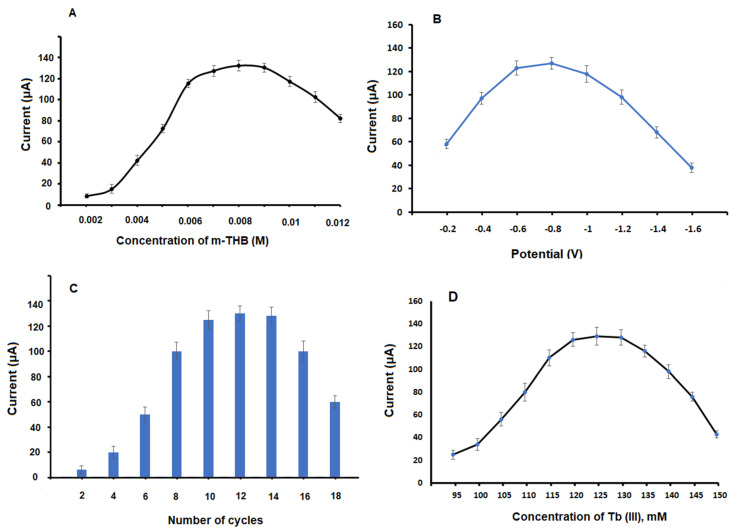
The effects of: (**A**) concentration of m-THB, (**B**) potential used in the polymerization of m-THB, (**C**) number of cycles for polymerization of m-THB and (**D**) concentration of Tb (III) on the current value of FAV (70 × 10^−9^ M) using Tb_NPS_ @ poly m-THB/PGE.

The present research is the first to investigate the electrochemical performance of phloroglucinol; an m-trihydroxy benzene compound (m-THB); on the peak current value of FAV; an antiviral regimen for COVID-19. Since modification of carbon-based electrodes is a promising step to improve electrode surface area and enhance its electron transfer, the electrochemical polymerization of m-THB was performed, which covers the surface of PGE. All experimental variables influencing the polymerization step were examined, such as m-THB concentration, potential, number of polymerization cycles and the scan rate. The concentration of m-THB has a great effect on the polymerization procedure and on the FAV current value. So, concentrations of m-THB from 0.002 to 0.012 M were studied, where the current value was increased by increasing the concentration till constant values of current were obtained using concentrations from 0.007 to 0.009 M, after that, a decrease in current value was found as the resultant polymer film using higher concentration may block the surface of the formed electrode and hence decrease current intensity (Figure 1A). Hence, 0.008 M was selected as the optimum concentration of m-THB and was subsequently used for further measurements. After that, the potential range necessary for m-THB polymerization was tested using different potential values ranging from −0.2 to −1.6 V. An increase in the current value of FAV was obtained by decreasing the potential value until a fixed current value was found at −0.8 V, and it was selected for further electrochemical measurements as represented in Figure 1B.

Owing to the importance of the number of cycles in the polymerization step, as it significantly affects electrode electro-catalytic activity, various numbers of cycles were performed. From data represented in Figure 1C, it was found that 12 cycles showed the highest values and a further decrease in current value was obtained using the number of cycles higher than 12. The scan rate was further tested from 0.05 to 0.4 V s^−1^, and the highest peak current value was observed at 0.1 V s^−1,^ as represented in Figure S1A (Supplementary Materials).

The formed polymer layers, poly m-THB over the PGE surface, were used as a platform for further loading of Tb _NPS_, where an electro-deposition of Tb (III) solution was performed. Different concentrations of Tb (III) ranging from 95 to 150 mM were examined; it was observed that the highest and most stable results were obtained using 125 mM, as shown in Figure 1D. Further, deposition potential values required from −1.8 to −0.4 V and various deposition time from 10 to 120 s was examined. It was noticed that −1.2 V and 80 s showed the highest values, and they were used as the optimum parameters, as shown in Figure S1B (Supplementary Materials).

### 3.2. Electrochemical Performance of FAV at Bare and Modified PGE

**Figure 2:**
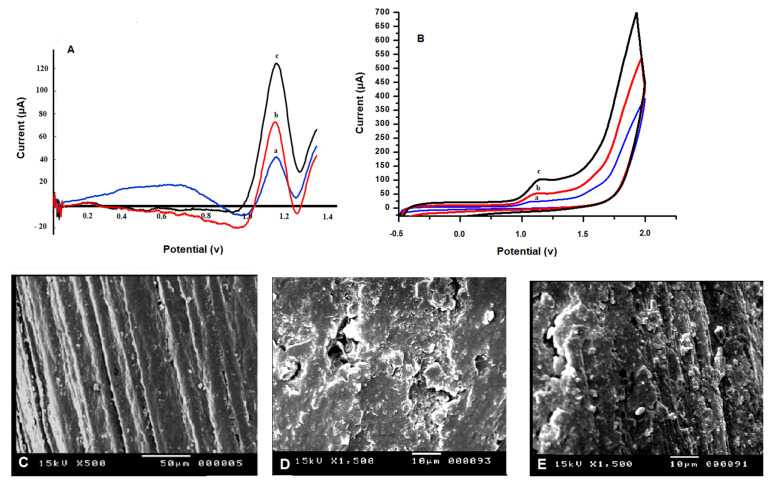
(**A**) SWVs, (**B**) CVs curves of FAV (70 × 10^−9^ M) recorded on: (a) bare PGE, (b) poly m-THB/PGE and (c) Tb_NPS_@ poly m-THB/PGE & SEM images of: (**C**) bare PEG, (**D**) poly m-THB/PGE, and (**E**) Tb _NPS_@ poly m-THB/PGE surface.

The electro-oxidation of FAV was established at bare and modified PGE by both CV and SWV techniques. Figure 2A shows the distinct oxidation peak of FAV (70 × 10 ^−9^ M) at potential 1.14 V using bare PGE (curve a), poly m-THB/PGE (curve b) in 0.1 M phosphate buffer, pH 7.0. The oxidation current values of FAV were 45 and 75 μA, respectively. Furthermore, Figure 2A (curve c) represented the deposition of Tb nanocomposites over poly m-THB/PGE surface, which remarkably enhanced the sensitivity towards FAV electro-oxidation, giving a higher current value (~127 μA) in comparison with bare PGE or poly m-THB/PGE which may be attributed to their good electro-activity, large surface areas and rapid transfer rate on the modified electrode surface. These findings clearly confirm the synergistic effect of the used hybrid composites, m-THB polymer and Tb _NPS_, in electrode composition.

### 3.3. Characterization of Tb_NPs_@ Poly m-THB/PGE

The morphological characterization of the fabricated electrodes was carried out by SEM technique, where SEM images of poly m-THB and Tb_NPs_@ poly m-THB were represented in Figure 2. The SEM images of the bare PGE electrode are represented in Figure 2C, where distinguishable smooth layers [32] covering its surface can be observed. However, after polymerization with m-THB, obvious lumps and flake-like structures were observed coating the surface of the modified electrode, as shown in Figure 2D. After incorporation of Tb_NPS_ over the surface of poly m-THB/PGE electrode, characteristic lumps and depressions structures with glowing clusters were observed over the surface of the modified electrode Tb_NPS_ @ poly m-THB/PGE electrode as shown in Figure 2E.

The used modification forms porous structures covering the electrode surface, which improves the active surface area of the electrode and enhances FAV oxidation. Moreover, the XRD pattern showed diffraction peaks centered around values of 28.76_and 47.50, which are in agreement with the diagnostic peaks of Tb, as shown in Figure S2A.

Figure S2B (Supplementary Materials) represents the UV spectra of m-THB, Tb and Tb_NPs_@ poly m-THB hybrid composite. As shown by curve i, a distinguished maximum peak of m-THB was observed at 268 nm. In addition, a characteristic peak of Tb was found at ~220 nm, as represented in curve ii. In curve iii (spectrum of Tb_NPs_@ poly m-THB hybrid modifier), another peak appeared at ~330 nm, which confirms the success of polymerization of m-THB, besides the characteristic peak of Tb at ~220 nm.

### 3.4. Investigation of Scan Rate

**Figure 3:**
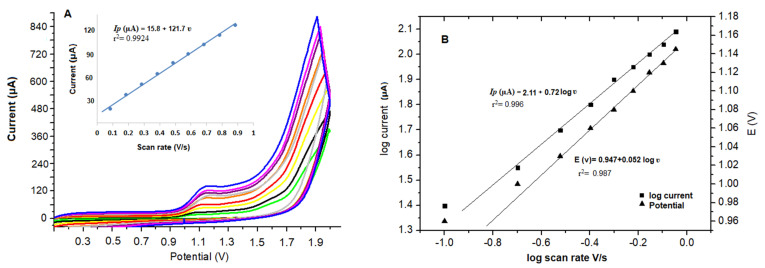
(**A**) The effect of different scan rates from 0.1 to 0.9 V s^−1^ on CVs curves of FAV (70 × 10^−9^ mol L^−1^), Inset: Scan rate calibration plot and (**B**) Dependence of logarithm peak current (*Ip*/µA) and the oxidation peak potential (*E*/V) on the logarithm of scan rate (log *ν*/Vs^−1^).

The investigation of the scan rate on the oxidation peak current of FAV in phosphate buffer (0.1 M, pH 7.0) using the fabricated electrode was performed by examining the related CV voltammograms at various scan rates from 100 to 900 mV/s. The peak current values of FAV were directly proportional to the scan rate, as shown in Figure 3, conforming Randles-Ševćik equation [39]. Figure 3A represents the enhancement in the anodic peak current by increasing the scan rate from 100 to 900 mV/s. The relationship between the oxidation peak current (*Ip*) and the scan rate (ʋ) showed a linear response following the equation below:

*Ip* (µA) = 15.8 + 121.7 ʋ   (r^2^ = 0.9924)

From Figure 3A, by increasing the scan rate, the oxidation potential of FAV was moved to more positive values, with an increase in the current intensity ensuring the irreversibility of the oxidation process of FAV. From data represented in Figure 3B, a plot of the logarithm of the oxidation peak current (log *Ip*) versus the logarithm of the scan rate (log ʋ) was found to be linear and was described by the following regression equation:

log *Ip* (µA) = 2.11 + 0.72 log ʋ(V s^−1^)  (r^2^ = 0.996)

The slope of the linear equation between log *Ip* versus log ʋ was 0.72. Hence the oxidation process of FAV is controlled by both adsorption and diffusion mechanisms, which agrees with the previous studies [18,19,20,21].

Further, by plotting potential (*Ep*) against the logarithm of scan rate (log ʋ), a linear relationship was obtained as represented in Figure 3B and described with the following regression equation:

E_p_ (V) = 0.947 + 0.052 log ʋ (V s^−1^)  (r^2^ = 0.987)

As represented in Figure 2B, no cathodic peak for FAV was observed in the CV reverse scan, confirming the irreversibility of the oxidation reaction of FAV. Based on the Laviron equation [40], the potential (E), number of transferred electrons (n) and scan rate in the rate-limiting step can be calculated from:

The slope of the plot E_p_ (V) and log ʋ = 2.2303 RT/αn F

T is the absolute temperature (298 K), n is the number of transferred electrons in the rate-determining step, R is the universal gas constant (8.314 J mol^−1^ K^−1^) and F is the Faraday constant (96.480 C mol^−1^). Assuming α (the transfer coefficient) is 0.5 in totally irreversible reactions, and after substitution of the slope with 0.052, the number of electrons involved in the oxidation process was calculated to be ≈2.0; this agrees with previously reported articles [20,21]. The oxidation reaction of FAV probably occurs in the aromatic hydroxyl group on the pyrazine ring in the FAV chemical structure, as mentioned before [19,20].

### 3.5. Electrochemical Characterization of Tb_NPs_@ Poly m-THB/PGE

**Figure 4:**
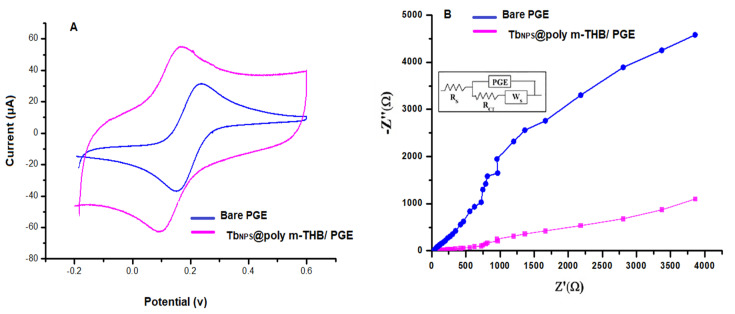
(**A**) Cyclic voltammograms and (**B**) Nyquist plot of 1.0 mM of Fe^3+^/Fe^2+^ solution recorded on bare PGE and Tb_NPS_@ poly m-THB/PGE electrodes. Working parameters are: scan rate 50 mV s^−1^ over a potential range of −0.2–0.6 V.

The electrochemical activity of Tb_NPs_@ poly m-THB/PGE was examined using the CV technique, where Fe^2+^/Fe^3+^ solution (1.0 mmol L^−1^) prepared in 0.5 M of potassium chloride was used. After using the modified sensor, an increment in peak current intensity in the reduction-oxidation peak of the Fe^2+^/Fe^3+^ system was observed, as represented in Figure 4A. This increment is due to an increase in the active surface area of the electrode after modification. This finding was affirmed by the EIS study, where measurements were performed at 10 mV, and the potential amplitude was within the frequency range (1.0–10 KHz). Tb_NPs_@ poly m-THB/PGE electrode showed lower series resistance than that of the bare one. Moreover, the results in Figure 4B shows that bare PGE has displayed a semi-circular model, but Tb_NPs_@ poly m-THB/PGE modified electrode displayed a straight linear curve. This indicates the improvement in the electrical conductivity due to the charge transfer acceleration and the surface area enlargement. In order to confirm this hypothesis, the electrode’s active surface area was calculated using the Randles–Ševćik equation from the slope of anodic peak current (*I_p_*):

*I_pa_* = (2.69 × 10^5^) n^3/2^A_eff_D_R_^1/2^C_0_υ^1/2^

A_eff_ is the electrode surface area in cm^2^, n is the number of electron transfers, D_R_ is the diffusion coefficient (cm^2^ s^−1^), C_0_ is the concentration of Fe^2+^/Fe^3+^ system (mol/cm^2^) and υ is the scan rate (V s^−1^). According to the Randles–Ševćik equation, the active surface areas of bare PGE and Tb_NPs_@ poly m-THB/PGE have been calculated to be: 0.212 and 0.454 cm^2^. This finding confirmed the improvement action of using the hybrid modification (m-THB polymer layers and Tb_NPS_) regarding the oxidation of FAV using a Tb_NPs_@ poly m-THB/PGE electrode.

### 3.6. Optimization of Method’s Parameters

#### 3.6.1. Effect of pH

Supporting electrolyte pH is very important to study the electrochemical behavior of FAV at the modified electrode. Therefore, different phosphate buffer solutions (0.1 M) from pH 4.0 to 9.0 were examined. An increase in the current value is observed by increasing the pH value. A phosphate buffer of pH 7.0 showed the highest value, and it was selected for further measurements, as represented in Figure S3A (Supplementary Materials). FAV molecule has two tautomeric forms, a more stable enol form and a ketone form, and the intersection point of the Ep/pH curves with a clear change in the peak intensity at about 7.0–8.0 may be explained by the replacement of one mechanism (reaction with enol) by another (reaction with ketone) in the tautomeric equilibrium of FAV. A linear plot between the pH values and their corresponding potential was represented in Figure S3B (Supplementary Materials), where the potential of the FAV oxidation peak was moved to less positive potential values upon increasing the pH value. It is noteworthy that Ep (V) = 1.122 + 0.053 pH (r = 0.994), which reveals the proton-dependent nature of FAV on the modified electrode. The value of the slope is close to the theoretical value of 59 mV; hence the number of electrons and protons involved in the electrochemical oxidation of FAV are equal. These findings are in agreement with the number of electrons (≈ 2.0 electrons) included in the oxidation of FAV, which is calculated above using the Laviron equation, and also in agreement with previously reported methods [19,20,21,22].

#### 3.6.2. SWV Parameters

Instrumental parameters affecting the proposed SWV, such as pulse height, step height and frequency, were studied in the ranges 3–30 mV, 1–30 mV and 25–250 V s^−1^, respectively. It was observed that pulse height at 5 mV, step height at 3 mV and frequency at 100 V s^−1^ were the optimal variables for electro-oxidation of FAV at the modified electrode. Besides, the initial potential and deposition time parameters of FAV were examined from −1.2 to + 0.8 V and from 10 to 90 s, and optimum values were −0.2 V and 60 s, respectively.

### 3.7. Validation Study

#### 3.7.1. Linearity and Sensitivity Limits

The calibration curve of FAV over a concentration range from 10–150 × 10^−9^ M was constructed under the optimum conditions as represented in Figure S4 (Supplementary Materials). The values of LOD and LOQ were 3.1 and 9.3 × 10 ^−9^ M, respectively. Various statistical parameters of the proposed SWV method are represented in Table 1. The proposed method showed higher sensitivity than various previously reported methods.

#### 3.7.2. Accuracy and Precision

The accuracy, repeatability and intermediate inter-day precisions for the proposed method were investigated. Table 2 summarizes the obtained results. The intra-day precision was evaluated by repeating the measurements of three various concentration levels of FAV working solutions (20, 70 and 120 × 10^−9^ M). The measurements were repeated over three consecutive days to examine the inter-day precision. Recovery results were observed to be from 98.92 to 101.42%, indicating acceptable accuracy. In addition, the % RSD values were calculated, and the results were less than 1.8%.

#### 3.7.3. Selectivity

The effect of commonly co-existing interfering substances during FAV analysis was examined in order to evaluate the selectivity of the fabricated Tb_NPS_@ poly m-THB/PGE electrode. The concentration of each substance was tested at 10 folds the concentration of the drug used during the electrochemical measurement. The percentage recovery values were in the range of 96.8–100.2% in the presence of various substances ensuring high selectivity of the proposed SWV method for FAV estimation (Table 3).

### 3.8. Method Applications

#### 3.8.1. Estimation of FAV in Pharmaceutical Tablet

The fabricated Tb_NPS_@ poly m-THB/PGE electrode showed high sensitivity (LOD = 3.1 × 10 ^−9^ M); hence it was successfully used to determine FAV in commercial tablets. Acceptable recovery results were obtained, ranging from 99.58–100.93% ± 1.2–1.9, ensuring the absence of interference from commonly used excipients, as represented in Table 4. The obtained percentage recovery results were statistically compared to those calculated according to a previously published method [7]. The % recovery of the proposed method was 98.9 ± 1.2 when compared with that of the reported one (97.5 ± 1.6). Moreover, the calculated values of the *t-* and *F*-tests (*n* = 5) were 0.63 and 2.35, respectively, ensuring a lack of significant difference between the proposed and the published methods.

#### 3.8.2. Estimation of FAV in Human Plasma

The fabricated electrode Tb_NPS_@ poly m-THB/PGE was employed for FAV quantitation in human plasma, where a calibration curve of human plasma samples spiked with FAV was constructed in concentrations ranging from 15 to 90 × 10^−9^ M to assess linearity. A good linearity was obtained with a correlation coefficient equal to 0.9994. The regression equation is Y *=* 0.81 *+* 2.04 X (*n* = 3); Y is the current value, and X is FAV concentration.

Moreover, % recovery was calculated, and the results ranged from 97.39 to 101.93% with RSD% values less than 2.5, as represented in Table 4. The acceptable results confirm the efficiency of the established SWV method utilizing Tb_NPS_@ poly m-THB/PGE for the detection of FAV without interferences from plasma constituents. The results obtained confirm the method’s sensitivity and applicability for the determination of FAV in human plasma samples.

### 3.9. Stability and Reproducibility of the Fabricated Sensor

The stability of the modified Tb_NPS_@ poly m-THB/PGE electro-sensor was examined, where the sensor was stored at 4 °C for 20 successive days, and the electrochemical behavior of FAV was assessed every 2 days. The storage stability of Tb_NPS_@ poly m-THB/PGE was illustrated by the measured current (Figure S5A, Supplementary Materials) with a 98.53% retention of the original, optimized current values. Moreover, FAV (70 × 10^−9^ M) was analyzed using six parallel-prepared modified electrodes fabricated using the same procedure. The corresponding RSD% value didn’t exceed 2.1% during the analysis, which proves the good reproducibility of the modified sensor, as shown in Figure S5B, Supplementary Materials.

The presented SWV method showed simplicity, good selectivity and high sensitivity utilizing the novel electrode (Tb_NPS_@ poly m-THB/PGE) in comparison with previously published articles, as represented in Table 5. Some of the reported methods suffered from several demerits like insufficient sensitivity, complicated procedures or the need for large amounts of organic solvents. This confirms the excellent applicability of the proposed SWV for drug analysis in different media.

## 4. Conclusions

An innovative and highly sensitive electrochemical sensor was fabricated based on utilizing Tb nanoparticles supported over new polymer layers of m-THB. The performance of the fabricated sensor was examined for studying the electro-oxidation of FAV using the SWV technique. CV, SEM, XRD, UV-spectrophotometry and EIS techniques were conducted for further characterization. Tb_NPs_ and poly m-THB had synergistic effects, which enhanced the determination of FAV in tablets and in human plasma. A full validation study of the proposed method was carried out according to the ICH guidelines. The proposed sensor showed good recovery results for the demonstration of FAV in different matrices without any interference. Besides, it has high sensitivity and simplicity in fabrication, and this ensured good performance of fabricated senor in FAV determination in complex matrices. Notably, this study is the first to be reported for the investigation of the electrochemical performance of the modified electrode (Tb_NPS_ @poly m-THB/PGE) in the analysis of FAV.

## Figures and Tables

**Table 1:** Linearity results for FAV determination by the proposed SWV method using Tb_NPS_@ poly m-THB/PGE electrode.

Parameter	FAV
Linearity range (×10^−9^ M)	10–150
Correlation coefficient (r)	0.9994
Intercept (a) ± SD *	1.46 ± 2.33
Slope (b) ± SD *	2.5 ± 0.04
LOD ** (×10^−9^ M)	3.08
LOQ ** (×10^−9^ M)	9.32

* Average of four replicates. ** Detection (LOD) and quantification (LOQ) limits were calculated as 3.3 σ/b and 10 σ/b, respectively, where σ is the standard deviation of the intercept of the regression equation and b is the slope of the calibration curve.

**Table 2:** Accuracy and precision results for determination of FAV under the developed SWV method.

Authentic Drug	Conc. (×10^−9^ M)	Accuracy		Intra-Day Precision		Inter-Day Precision	
**FAV**	20	% Recovery *	%RSD *	% Recovery *	%RSD *	%Recovery *	% RSD *
		100.36	1.25	99.53	0.99	100.64	1.77
	70	98.92	0.93	101.42	1.81	101.32	1.34
	120	101.14	1.36	100.16	1.43	99.75	1.11

* Average of three replicates; Results are compared with those of the standard calibration curve.

**Table 3:** Selectivity of the proposed SWV method for determination of FAV.

Interfering Material	Inter-Day Precision	
	% Recovery *	% RSD *
**Uric acid**	99.3	1.2
**Oxalic acid**	100.2	0.9
**Sucrose**	97.3	1.1
**Starch**	99.1	1.4
**Magnesium chloride**	97.7	1.3
**Citric acid**	96.8	1.5
**L-Ascorbic acid**	97.2	1.7

* Average of three replicates; FAV: Interfering substance (1: 10).

**Table 4:** Determination of FAV in tablets and human plasma using Tb_NPS_@ poly m-THB/PGE electrode.

Sample	Added Amount (nM)	Found Concentration (nM)	%Recovery ^a^ ± %RSD	
Avipiravir^®^ Tablet	20	20.09	100.45 ± 1.2	
	80	78.87	98.58 ± 1.6	
	120	121.12	100.93 ± 1.9	
Human plasma	30	29.56	98.53 ± 2.1	
	50	50.07	100.14 ± 1.8	
	70	71.35	101.93 ± 1.5	
	120	116.87	97.39 ± 1.7	
Proposed method		Reported method [8]	t-value ^b^	F-Value ^b^
98.9 ± 1.2		97.5 ± 1.6	0.63	2.35

^a^ Average of three replicates. ^b^ Theoretical values at 95% confidence limit; t = 2.228, F = 5.053; (*n* = 6).

**Table 5:** Comparison between the proposed method for determination of FAV to previously reported methods.

Technique	Linearity Range	LOD	Application	Ref.
HPLC	0.5–50 µg/mL	0.04 µg/mL	Tablet	[5]
Spectrofluorimetry	40–280 ng/mL	9.44 ng/mL	Tablet/human plasma	[6]
Synchronous Fluorimetry	1–18 ng/mL	0.25 ng/mL	Tablet/human plasma	[8]
HPTLC	3.75–100 µg/mL	1.12 µg/mL	Pure form/Tablet	[11]
HPLC-UV	10–100 µg/mL	1.2 µg/mL	Tablet	[12]
HPLC-DAD	6.25–250 µg/mL	1.02 µg/mL	Tablet	[13]
UPLC-MS/MS	0.25–16 µg/mL	0.075 µg/mL	Human plasma	[14]
LC-MS/MS	0.048–50 µg/mL	0.045 µg/mL	Human serum	[17]
LC-MS/MS	0.1–20 µg/mL	0.03 µg/mL	Tablet &plasma	[15]
UPLC	0.1–10 µg/mL	0.03 µg/mL	Human plasma	[16]
SWV	0.01–0.1 µg/mL 0.1–20 µg/mL	3 ng/mL	Tablet/human urine	[18]
SWV	1.56–31.2 µg/mL	0.76 ng/mL	Tablet/serum	[19]
SWV	864–0.157 µg/mL	0.017 µg/mL	Tablet/urine	[22]
DPV	0.014–0.31 µg/mL	0.072 ng/mL	Tablet/plasma & urine	[20]
SWV	1–100 µg/mL	0.26 μg/mL	Tablet/urine	[21]
SWV	1.57–23.6 ng/mL	0.486 ng/mL	Tablets & human plasma	Proposed study

## Data Availability

All data are available from the corresponding author upon reasonable request.

## Supplementary Materials

The following supporting information can be downloaded at: https://www.mdpi.com/article/10.3390/bios13020243/s1, Figure S1: Effect of (A) scan rate on the current of FAV (70 × 10^−9^ M) in electro-polymerization process of m-THB, and (B) deposition potential of Tb (III) solution; Figure S2: (A) XRD spectrum of Tb nanoparticles and (B) Uv spectra of: (i) m-THB, (ii) Tb and (iii) poly m-THB& Tb hybrid composite; Figure S3: (A) The effect of pH of supporting electrolyte pH on the oxidation peak of FAV (70 × 10^−9^ M), and (B) Linear plot between potential and pH values of the supporting electrolytes; Figure S4: Square wave voltammograms for various concentrations of FAV (10–150 × 10^−9^ M) monitored on Tb_NPS_@ poly m-THB/PGE electrode.; Figure S5: Bar diagram curves obtained for (A) stability studies of Tb_NPS_@ poly m-THB/PGE modified electrode over 20 days and (B) recording six different times to attain reproducibility study of Tb_NPS_@ poly m-THB/PGE in the presence of FAV (70 × 10^−9^ M).
