# Supplementary material for: An Innovative Polymer-Based Electrochemical Sensor Encrusted with Tb Nanoparticles for the Detection of Favipiravir: A Potential Antiviral Drug for the Treatment of COVID-19
Source: Biosensors (Basel). 2023 Feb 8;13(2):243. doi: 10.3390/bios13020243 (PMC9954130; doi:10.3390/bios13020243)
Supplement: Supplementary file 1 [file biosensors-13-00243-s001.zip › biosensors-2155523-supplementary.pdf]

Supplementary Material

Article

An Innovative Polymer-based Electrochemical Sensor Encrusted with Tb nanoparticles for the Detection of Favipiravir; A potential antiviral drug for treatment of COVID-19

Marwa F. B. Ali[1], Roshdy E. Saraya[2], Sami El Deeb [3,4*], Adel Ehab Ibrahim[2,4], and Baher I. Salman[5]

[1] Department of Pharmaceutical Analytical Chemistry, Faculty of Pharmacy, Assiut University, Assiut 71526, Egypt
[2] Pharmaceutical Analytical Chemistry Department, Faculty of Pharmacy, Port-Said University, Port Said 42526, Egypt
[3] Institute of Medicinal and Pharmaceutical Chemistry, Teschnische Universität Braunschweig, 38106 Braunschweig, Germany
[4] Natural and Medical Sciences Research Center, University of Nizwa, Birkat Al Mauz, P.O. Box 33, Nizwa 616, Oman
[5] Pharmaceutical Analytical Chemistry Department, Faculty of Pharmacy, Al-Azhar University, Assiut Branch, Assiut 71524, Egypt
* Correspondence: s.eldeeb@tu-bs.de; Tel.: +49-531-391-7301

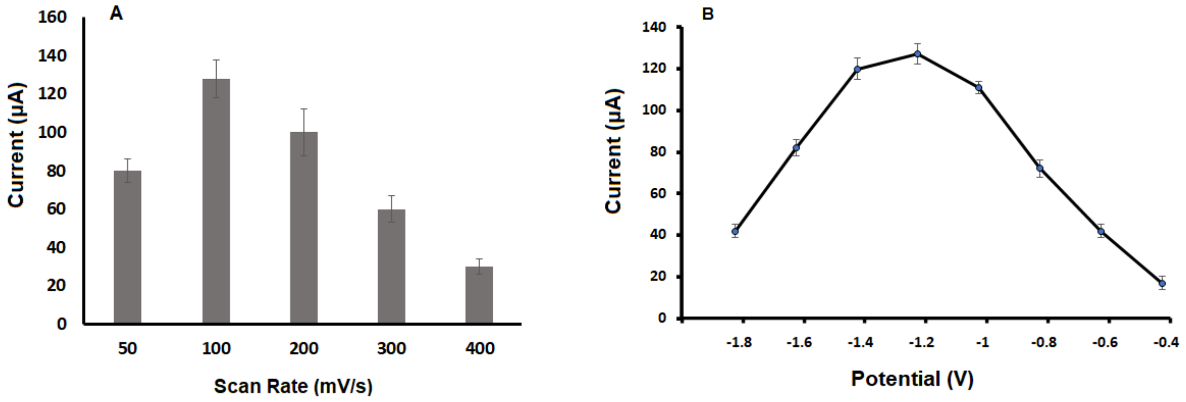

Figure S1. Effect of A) scan rate on current of FAV (70 x 10^{-9} M) in electro-polymerization process of m-THB, and B) deposition potential of Tb (III) solution.

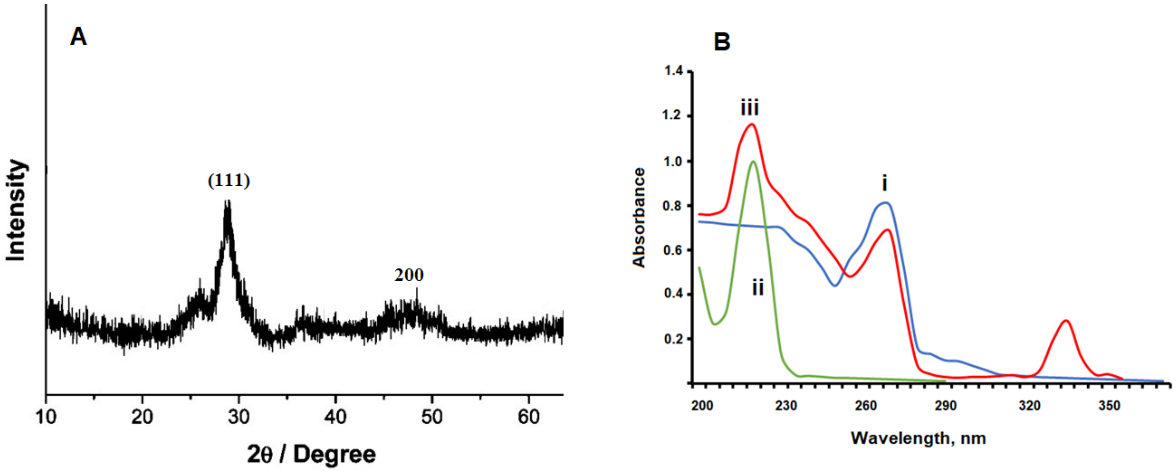

Figure S2. A) XRD spectrum of Tb nanoparticles and B) Uv spectra of: i) m-THB, ii) Tb and iii) poly m-THB& Tb hybride composite.

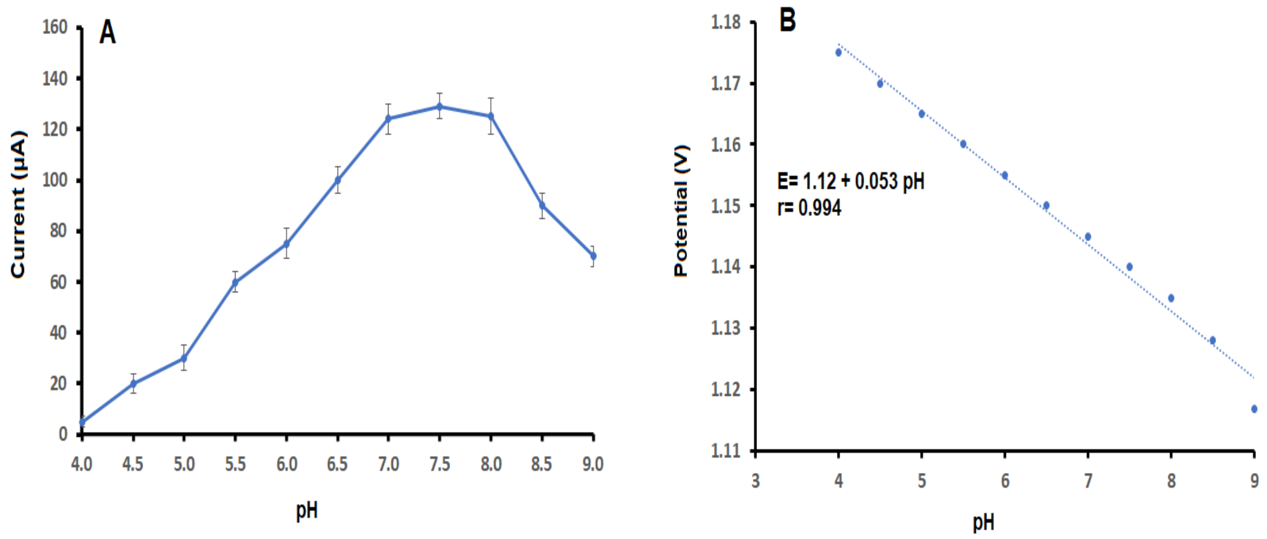

Figure S3. A) The effect of pH of supporting electrolyte pH on the oxidation peak of FAV (70 x10^{-9} M), and **B)** Linear plot between potential and pH values of the supporting electrolytes.

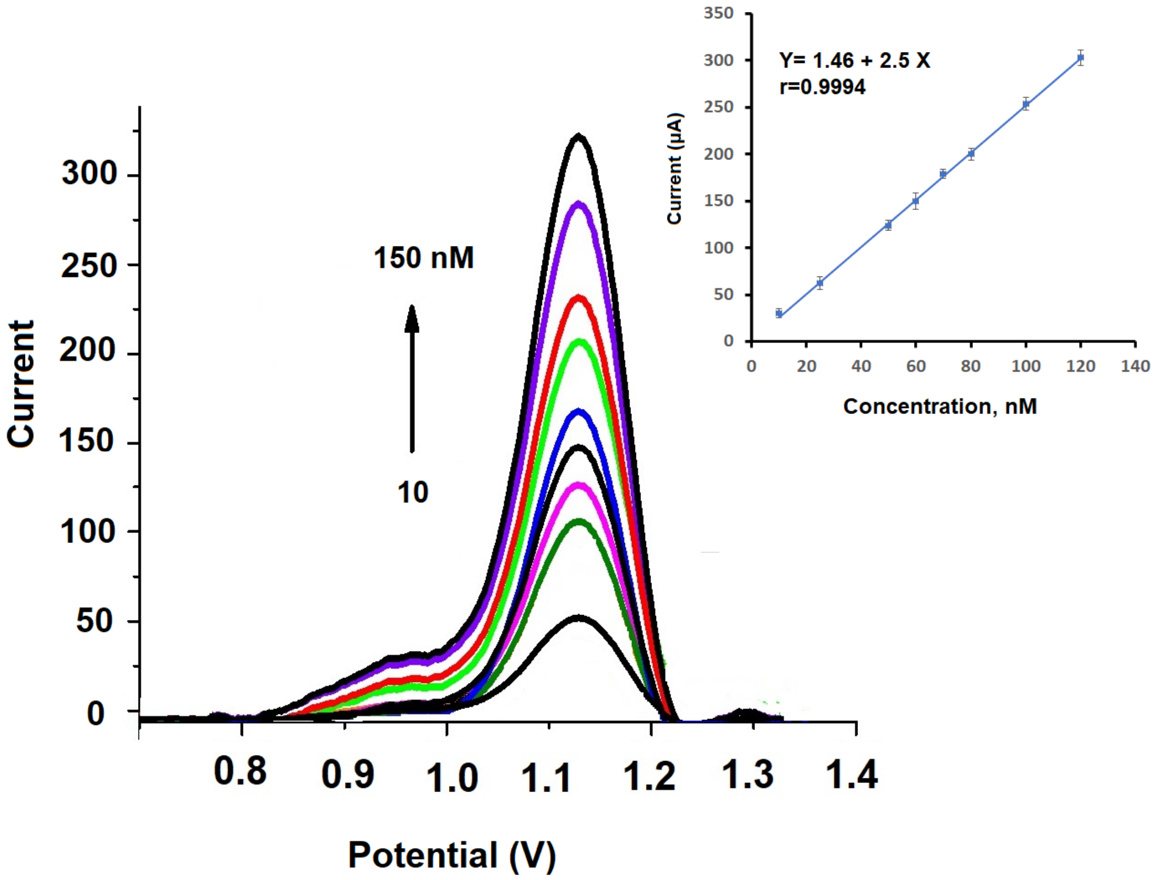

Figure S4. Square wave voltammograms for various concentrations of FAV (10-150 x 10^{-9} M) monitored on Tb_{NPS}@ poly m-THB/PGE electrode.

Optimum parameter: 0.1 M phosphate buffer (pH= 7.0), E_{acc}= -0.2 V, frequency=100 Hz, pulse height=5 mV, step height=3 mV, t_{acc}= 60 sec. Inset: calibration curve between concentration and peak current.

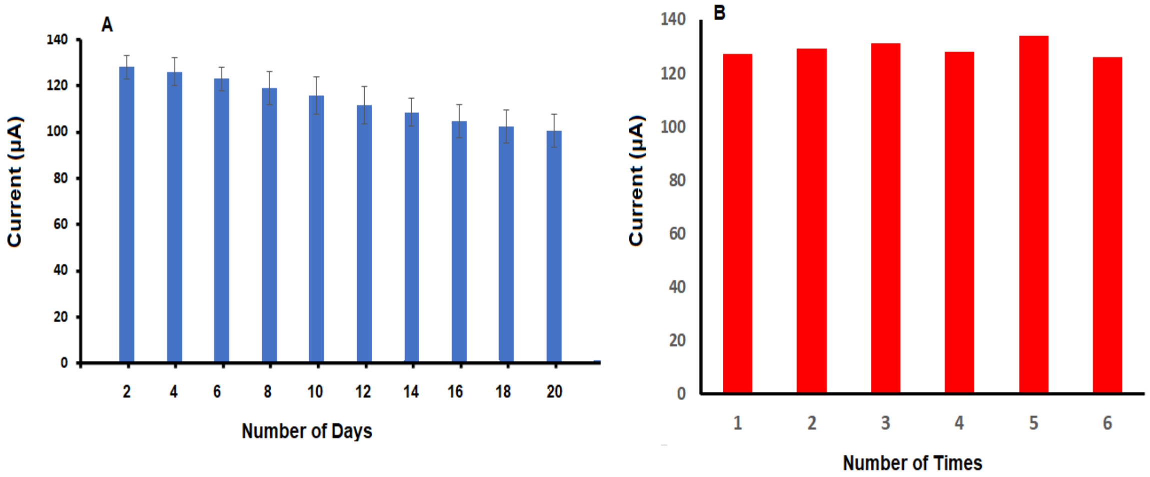

Figure S5. Bar diagram curves obtained for: A) stability studies of Tb$_{NPS}$@ poly m-THB/PGE modified electrode over 20 days and B) recording six different times to attain reproducibility study of Tb$_{NPS}$@ poly m-THB/PGE in the presence of FAV (70×10^{-9} M).
